# Antinociceptive Effects and Interaction Mechanisms of Intrathecal Pentazocine and Neostigmine in Two Different Pain Models in Rats

**DOI:** 10.1155/2022/4819910

**Published:** 2022-05-18

**Authors:** Huiying Huang, Xiaohui Bai, Kun Zhang, Jin Guo, Shaoyong Wu, Handong Ouyang

**Affiliations:** ^1^Department of Blood Transfusion, Guangzhou First People's Hospital, School of Medicine, South China University of Technology, Guangzhou 510180, Guangdong, China; ^2^Department of Anesthesiology, State Key Laboratory of Oncology in Southern China, Sun Yat-sen University Cancer Center, Collaborative Innovation Center for Cancer Medicine, Guangzhou, China; ^3^Department of Anesthesiology, Guangdong Provincial Key Laboratory of Malignant Tumor Epigenetics and Gene Regulation. Sun Yat-sen Memorial Hospital, Sun Yat-sen University, Guangzhou, China

## Abstract

**Background:**

Pentazocine produces a wide variety of actions in the treatment of perioperative analgesia. Neostigmine is a cholinesterase inhibitor used to antagonize the residual effects of muscle relaxants and also produces an analgesic effect.

**Objectives:**

To investigate the analgesic effects of intrathecally injected pentazocine and neostigmine and their interaction.

**Methods:**

Sprague–Dawley rats were used to test the analgesic effect of pentazocine and neostigmine using the paw formalin pain model and the incision mechanical allodynia model. Pentazocine (3, 10, 30, and 100 *μ*g), neostigmine (0.3, 1, 3, and 10 *μ*g) or a pentazocine-neostigmine mixture were separately injected to evaluate their antinociceptive effects alone on the treatment groups. The corresponding control group received an intrathecal injection containing the same volume of saline. The formalin pain test, or the plantar incision pain behavior test were performed 30 minutes later. Isobolographic analysis was used to evaluate the interaction between pentazocine and neostigmine. Intrathecally administered selective mu-opioid receptor antagonist CTAP, selective kappa-opioid receptor antagonist nor-Binaltorphimine (nor-BNI), nonselective opioid receptor antagonist naloxone, and muscarinic acetylcholine receptor antagonist atropine were also used to test the possible interaction mechanism. These antagonists were used 30 minutes before the pentazocine and neostigmine mixtures which were intrathecally injected.

**Results:**

Intrathecally administered pentazocine (3, 10, 30, and 100 *μ*g) and neostigmine (0.3, 1, 3, and 10 *μ*g) alone had a marked dose-related impact on suppressing the biphasic responses in the formalin test. Pentazocine (3, 10, 30, and 100 *μ*g) and neostigmine (0.3, 1, 3, and 10 *μ*g) alone attenuated the mechanical allodynia in a plantar incision model in a dose-dependent manner. Isobolographic analysis revealed that the mixture of intrathecal pentazocine and neostigmine synergistically decreased both phase I and II activity in the formalin test and mechanical allodynia in the plantar incision model. Pretreatment of intrathecally administered nor-BNI, naloxone, atropine, but not CTAP, antagonized the analgesic effect of the pentazocine-neostigmine mixture.

**Conclusions:**

All of these results suggest that the combined application of pentazocine and neostigmine is an effective way to relieve pain from formalin and acute incision mechanical allodynia. The synergistic effect between pentazocine and neostigmine is mostly attributed to the kappa-opioid receptor and the cholinergic receptor in the spinal cord.

## 1. Introduction

Pentazocine was previously classified as a kappa-opioid receptor agonist and a mu-opioid receptor antagonist, but researchers later determined it as a mixed kappa-opioid receptor agonist and a partial mu-opioid receptor agonist [1–3]. It shares lots of the side effects of other opioids such as constipation and nausea, but it produces less central nervous system depression. While it seldom affects the mood after short-term use, it sometimes causes hallucinations, nightmares, and delusions [4]. Intravenously administered pentazocine can reduce both the incidence and severity of itching in women treated with subarachnoid opioids during cesarean section [5]. Pentazocine is also effective in alleviating postoperative pain and is commonly used as an analgesic in the perioperative period [6]. Three major opioid receptor systems, mu-opioid, delta-opioid, and kappa-opioid have been characterized with respect to the signal transduction pathway leading to pain modulation. Activation of the kappa-opioid system within the nucleus accumbens may circumvent pain-induced affective disorders [7], and *µ*-heterodimerization may be a potential target in a spinal nerve injury neuropathic pain model [8]. Spinal cord cholinergic receptors and acetylcholine (Ach) participate in the transmission and regulation of pain, and acetylcholine receptors have been identified as a target for pain control for decades [9, 10]. Ach is released in response to pain stimuli at the spinal cord and brain stem level [11]. Cholinesterase inhibitor physostigmine has been shown to relieve clinical postoperative pain [12]. And neostigmine, a clinical cholinesterase inhibitor, is widely used as an anesthetic to reverse nondepolarizing neuromuscular blockers. It is also regularly combined with atropine as atropine can block the muscarinic acetylcholine receptor [13]. Neostigmine produces a dose-dependent analgesic after spinal and peripheral administration in preclinical and clinical trials [14, 15]. It inhibits cholinesterase and results in much more ACh at sites of cholinergic transmission. Direct activation of cholinergic receptors or the pharmacological blocking of acetylcholinesterase to amplify endogenous acetylcholine action has been proven to alleviate pain in rodents and humans [9]. A reduction in cholinergic modulation may also be integral to mPFC deactivation for neuropathic pain, as well as underscore mPFC related cognitive shortfalls related to such pain [16]. In the process of anesthetic resuscitation, pentazocine is sometimes used as an analgesic, and neostigmine as a muscle relaxant antagonist. However, there is no research on the combined effects of pentazocine and neostigmine for postoperative pain treatment. In this experiment, the actual effects of pentazocine and neostigmine were assessed using a formalin-induced pain model and the plantar incisional pain model. To explore its possible mechanism of action, we intrathecally administered the selective kappa-opioid receptor antagonist nor-Binaltorphimine, the selective mu-opioid receptor antagonist CTAP, the nonselective opioid receptor antagonist naloxone, and the muscarinic acetylcholine receptor antagonist atropine.

## 2. Materials and Methods

### 2.1. Animals and Drug Administration

This research was approved by the Animal Care and Use Committee of Sun Yat-Sen University (No. L10202020000X, Guangzhou, China). Three hundred and eighty male Sprague–Dawley (SD) rats weighing 200 to 250 g were used. Rats were kept in separate cages with 50 to 60% humidity at 24°C with free access to food and water. All surgical procedures were performed with the rats under isoflurane (1–3%) inhalation. Nor-Binaltorphimine 10 *μ*g (nor-BNI, Abcam ab120078), CTAP 10 *μ*g (R&D, 1560/1), naloxone 10 *μ*g (Merck, 465-65-6), and atropine 10 *μ*g (Merck, 51-55-8) were administered 30 minutes before the pentazocine (Merck, 359-83-1)-neostigmine (Merck, 114-80-7) mixture.

### 2.2. Drugs

The drugs nor-Binaltorphimine 10 *μ*g, CTAP 10 *μ*g, naloxone 10 *μ*g, atropine 10 *μ*g, pentazocine (3–100 *μ*g), neostigmine (0.3–10 *μ*g), and pentazocine-neostigmine were respectively dissolved in 10 *μ*l of normal saline and were administered intrathecally [17–20]. The antagonists were intrathecally administered as described elsewhere [21].

### 2.3. Treatment Schedule and Experimental Design

In this study, we designed four independent experiments, and our study established *n* = 10 rats for each experimental group. All the rats were randomly divided into different groups.

**Figure 1:**
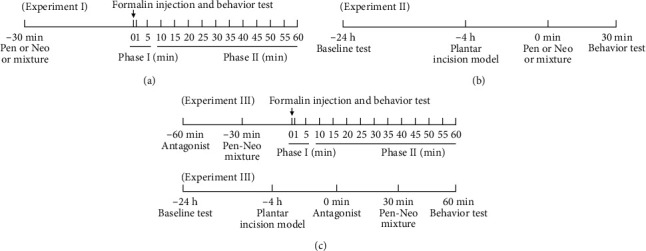
Treatment schedule.

Experiment I: Male SD rats (*n* = 130) were divided into 13 groups and intrathecally administered (i.t.) 30 minutes before the formalin test with different doses of pentazocine (3 *μ*g, 10 *μ*g, 30 *μ*g, and 100 *μ*g, i.t., *n* = 10), neostigmine (0.3 *μ*g, 1 *μ*g, 3 *μ*g, and 10 *μ*g, i.t., *n* = 10), saline alone (10 *μ*l, i.t., *n* = 10), or the pentazocine-neostigmine mixture (1/2ED_50_, 1/4ED_50_, 1/8ED_50_, and 1/16ED_50_, i.t., *n* = 10) (Figure 1(a)).

Experiment II: Male SD rats (*n* = 130) were divided into 13 groups and intrathecally administered (i.t.) 4 hours after a plantar incision model with different doses of pentazocine (3 *μ*g, 10 *μ*g, 30 *μ*g, and 100 *μ*g, i.t., *n* = 10), neostigmine (0.3 *μ*g, 1 *μ*g, 3 *μ*g, and 10 *μ*g, i.t., *n* = 10), saline alone (10 *μ*l, i.t., *n* = 10), or the pentazocine-neostigmine mixture (1/2ED_50_, 1/4ED_50_, 1/8ED_50_, and 1/16ED_50_, i.t., *n* = 10) (Figure 1(b)).

Experiment III: Male SD rats (*n* = 120) were divided into 12 groups and several antagonists were intrathecally administered (i.t.) 30 minutes before the administration of the pentazocine (30 *μ*g)-neostigmine (3.0 *μ*g) mixture. Saline alone (10 *μ*l), nor-Binaltorphimine (10 *μ*g), CTAP (10 *μ*g), naloxone (10 *μ*g), and atropine (10 *μ*g), were intrathecally administered 30 minutes before the administration of the pentazocine (30 *μ*g)-neostigmine (3.0 *μ*g) mixture in the formalin test and incision pain model (Figure 1(c)).

### 2.4. Intrathecal Injection

Under isoflurane (1–3%) inhalation anesthesia, a sterile needle attached to a 25 *μ*l micro-injector was inserted into the intervertebral space between L5 and L6 in rats. A sudden and slight flick of the tail indicated that the needle entered the subarachnoid space, where 10 *μ*l of the specific therapeutic drug or vehicle was delivered for more than 30 seconds [21]. The needle was held in the same position for an additional 15 seconds to ensure diffusion prior to removal. Thirty minutes after intrathecal injection the behavioral tests were performed.

### 2.5. The Formalin Test

A 30-gauge needle was used to inject 50 *μ*l of 5% formalin subcutaneously into the plantar surface of the left hind paw [17]. Next, the rats were placed in a transparent organic glass cylinder (20 cm × 30 cm) for observation. A mirror was placed under the cylinder at a 45° angle. Immediately after injection, the rat exhibited the behavioral plantar pain phenomenon exhibiting spontaneous flinching, withdrawing, and licking of the injected paw. Pain behavior was quantified by recording the number of paw flinches for 1-minute periods from 1 to 2 minutes and 5 to 6 minutes and then at 5-minute intervals between 10 minutes and 60 minutes after the formalin injection. We observed two phases of paw flinching behavior. The first stage in the pain model (the interval between 0 and 6 minutes after the formalin injection) is the initial acute pain response, followed by the second stage, persistent pain (starting about 10 minutes after the formalin injection).

### 2.6. The Plantar Incision Model

In order to simulate acute postoperative pain, we used the rat plantar incision model [22]. The rats were anesthetized with 2–3% isoflurane. Make a 1 cm longitudinal incision at the plantar surface of the right hind foot 0.5 cm from the heel end. The skin, fascia, and underlying flexor muscles were cut, and the wound was sutured with 5–0 nylon sutures after sufficient hemostasis. The sham control group rats were anesthetized without incision. Four hours after the pain model was established, the rat behavior test was performed.

### 2.7. Mechanical Allodynia

Mechanical allodynia (other pain) is a painful sensation triggered by an innocuous stimulus, such as a light touch [23]. Rats were placed on a mesh floor individually covered with a clear plastic cage and allowed to acclimate for 30 minutes. Paw withdrawal response to mechanical stimulation was detected by the calibrated von Frey hairs method. Mechanical sensitivity was assessed using the von Frey hairs (0.4 g, 0.6 g, 1 g, 2 g, 4 g, 6 g, 8 g, 10 g, and 15 g) up-down method as previously described [24].

### 2.8. Isobolographic Analysis

To test the interaction between pentazocine and neostigmine, we performed an isobolographic analysis. First, each ED_50_ value (effective dose producing a 50% maximal possible effect) was identified by the dose-response curves for each of the two drugs. In the formalin test, the time response data are presented as the number of paw flinches in the 1-minute time frames of 1 to 2 minutes and 5 to 6 minutes in the first phase and then at 5-minute intervals during the period from 10 minutes to 60 minutes in the second phase. In order to get the ED_50_, the flinches were converted into a percentage maximal possible effect (%MPE). We defined separately the value of 50%MPE as ED_50_ calculated by the following formula in the two stages of formalin test:

(1) $$\begin{array}{c} \text{\%MPE}=\frac{\text{Sum of control phase I }\left(\text{II}\right)\text{ count}-\text{Sum of phase I }\left(\text{II}\right)\text{ count with drug}}{\text{Sum of control phase I }\left(\text{II}\right)\text{ count}}*100. \end{array}$$

In the plantar incision model, the 50% MPE (ED_50_) was calculated using the following formula [25]:

(2) $$\begin{array}{c} \text{\%MPE}=\frac{\text{Threshold post drug}-\text{Threshold post control}}{\text{Baseline}-\text{Threshold post control}}*100. \end{array}$$

Next, the respective ED_50_ values (1/2, 1/4, 1/8, and 1/16) for each drug were coadministered. The experimental ED_50_ for the mixture was calculated by the dose-response curves of the mixture. The expected additive ED_50_ values for pentazocine and neostigmine were determined by an isobologram. The *x* and *y* axes in the isobologram represent the ED_50_ values of each drug, respectively. The lines connecting the ED_50_ points are the theoretical additive lines and the theoretical additive points for the drug combinations. The experimental values below the lines of additivity indicate a synergistic interaction [25].

### 2.9. Statistical Analysis

All data were expressed as the means ± SEM. The Shapiro–Wilk test was used to detect the normality of the data distribution. To compare the differences between each dose of pentazocine, neostigmine, and the mixtures of pentazocine and neostigmine in two stages of the formalin test and the von Frey test, we used one-way analysis of variance (ANOVA) or two-way ANOVA followed by the Bonferroni post hoc test. The differences between the experimental ED_50_ for pentazocine, neostigmine, and the mixtures, and the expected additive ED_50_ values for pentazocine, neostigmine, and the mixtures were evaluated by the one-way ANOVA in the formalin test and the von Frey test. The criterion for statistical significance was a *P* < 0.05. Statistical tests were performed with SPSS 21.0 software (SPSS, USA).

## 3. Results

### 3.1. The Effects of Pentazocine and Neostigmine on Formalin-Induced Pain

**Figure 2:**
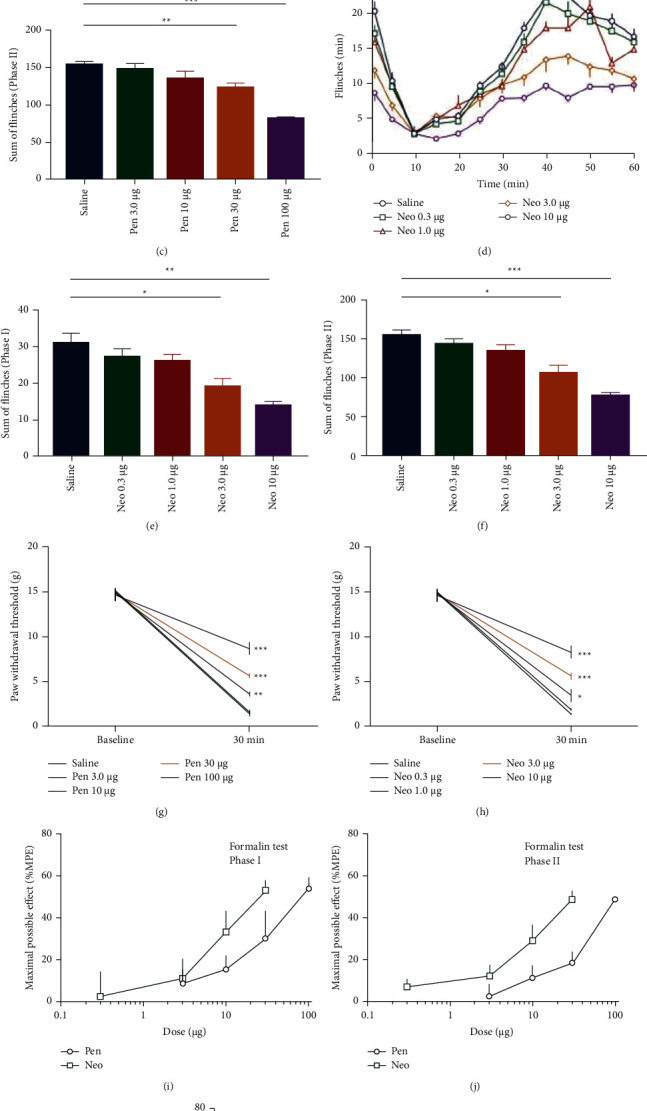
(a-f). Intrathecal injection of pentazocine (3–100 *μ*g) and neostigmine (0.3–10 *μ*g) induced a dose-dependent inhibition against the formalin-induced pain responses in both phases. (g-h). Injecting pentazocine or neostigmine attenuated the plantar incision-induced mechanical allodynia. (i-k). Dose-response curves of intrathecal pentazocine and neostigmine for flinching during phase I (i) and phase II (j) in the formalin test (k). Data are expressed as the maximal possible effect (% MPE). Each point on the graph represents the mean ± SEM. Compared with the saline group, ^*∗*^*P* < 0.05, ^*∗∗*^*P* < 0.01, ^*∗∗∗*^*P* < 0.001, *n* = 10 rats in each group).

The number of flinches for each minute is plotted versus the time after the formalin injection into the hind paw. Two phases of paw flinching behavior were separately quantified. Intrathecal administration of pentazocine (Pen) and neostigmine (Neo) decreased the number of paw flinches. Pentazocine (3–100 *μ*g) relieved the formalin-induced phase I pain (one-way ANOVA, *F*_4,45_ = 9.990, *P* < 0.001) (Figure 2(b)) and phase II pain (one-way ANOVA, *F*_4,45_ = 20.675, *P* < 0.001) (Figure 2(c)). Neostigmine (0.3–10 *μ*g) also relieved the formalin induced phase I pain (one-way ANOVA, *F*_4,45_ = 10.649, *P* < 0.001) (Figure 2(e)) and phase II pain (one-way ANOVA, *F*_4,45_ = 16.748, *P* < 0.001) (Figure 2(f)). In phases I and II, pentazocine's calculated ED_50_ values were 91.1 ± 5.4 *μ*g and 101.2 ± 6.2 *μ*g, respectively. Neostigmine's ED_50_ values in phases I and II were 8.93 ± 0.59 *μ*g and 9.2 ± 0.55 *μ*g, respectively (Figures 2(i) and 2(j), Table 1).

### 3.2. The Effects of Pentazocine and Neostigmine on Plantar Incision-Induced Mechanical Allodynia

Behavior was tested 30 minutes after the intrathecal injection. Intrathecal injection of pentazocine (3–100 *μ*g) produced a dose-dependent inhibition against the plantar incision-induced mechanical allodynia (repeated measure, two-way ANOVA, *F*_4,36_ = 29.178, *P* < 0.001) (Figure 2(g)). Intrathecal injection of neostigmine (0.3–10 *μ*g) produced a dose-dependent inhibition against the plantar incision-induced mechanical allodynia (repeat measure, two-way ANOVA, *F*_4,36_ = 23.751, *P* < 0.001) (Figure 2(h)). The calculated ED_50_ values of pentazocine and neostigmine were 98.0 ± 5.5 *μ*g and 10.2 ± 0.55 *μ*g, respectively (Figure 2(k), Table 1).

### 3.3. Isobolographic Analyses

**Figure 3:**
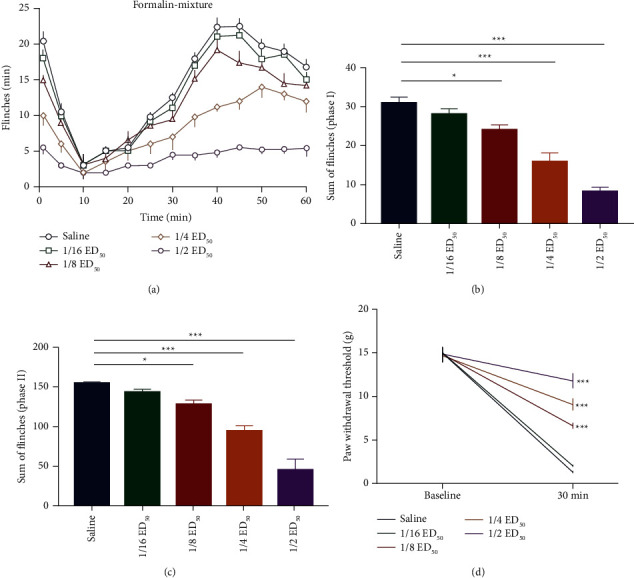
(a-c). Intrathecal administration of the pentazocine-neostigmine mixtures attenuated the number of paw flinches in the formalin pain model. (d). Intrathecal administration of the pentazocine-neostigmine mixtures attenuated the mechanical allodynia in the plantar incision pain model. Each bar represents the mean ± SEM from 10 rats. Compared with the saline group, ^*∗*^*P* < 0.05, ^*∗∗*^*P* < 0.01, ^*∗∗∗*^*P* < 0.001, *n* = 10 rats in each group.

We used the ED_50_ of pentazocine and neostigmine in phase I to evaluate their interaction in the formalin pain model. The ED_50_ of pentazocine and neostigmine was 91.1 *μ*g and 8.93 *μ*g in phase I, respectively. Intrathecal administration of the pentazocine-neostigmine mixtures (1/2 ED_50_, 1/4 ED_50_, 1/8 ED_50_, and 1/16 ED_50_) decreased the number of paw flinches (Figure 3(a)). In phase I, the pentazocine-neostigmine mixtures relieved the pain induced by formalin (one-way ANOVA, *F*_4,45_ = 34.687, *P* < 0.001) (Figure 3(b)). In phase II, the pain was relieved by the pentazocine-neostigmine mixtures (one-way ANOVA, *F*_4,45_ = 99.844, *P* < 0.001) (Figure 3(c)). In the plantar incision pain model, pentazocine ED_50_ was 98 *μ*g and neostigmine ED_50_ was 10.2 *μ*g. Intrathecal administration of the pentazocine-neostigmine mixtures (1/2 ED_50_, 1/4 ED_50_, 1/8 ED_50_, and 1/16 ED_50_) significantly attenuated the mechanical allodynia (repeat measure, two-way ANOVA, *F*_4,36_ = 50.640, *P* < 0.001) (Figure 3(d)).

**Figure 4:**
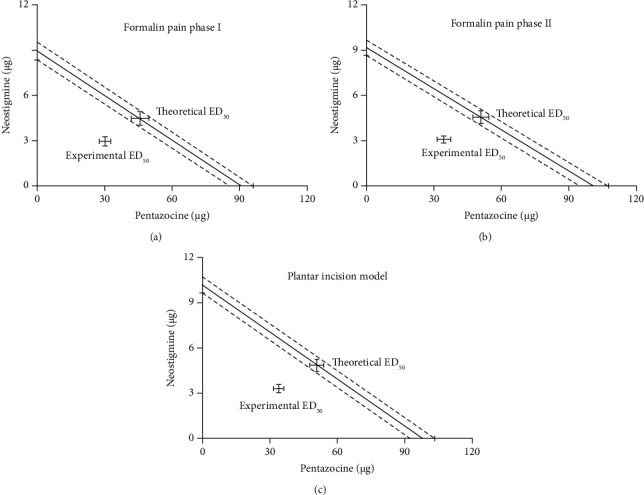
Shows the interaction between intrathecal pentazocine and neostigmine in phases I (a) and II (b) of the formalin tests and the plantar incisional pain model (c) which were analyzed using an isobologram. The *x*- and *y*-axes represent the ED_50_ dose of pentazocine and neostigmine, respectively. The lines connecting the ED_50_ points are the theoretical additive lines and the theoretical additive points for the drug combinations. The theoretical additive value was significantly higher than the experimental ED_50_ value of the combination of the two drugs. The experimental ED_50_ values were significantly below the lines of additivity, indicating a synergistic interaction.

The experimental ED_50_ for pentazocine and neostigmine were 30.3 ± 2.5 *μ*g and 2.95 ± 0.3 *μ*g in phase I, respectively (Figure 4(a)). The experimental ED_50_ for pentazocine and neostigmine were 34.4 ± 3.0 *μ*g and 3.1 ± 0.24 *μ*g in phase II, respectively (Figure 4(b)). The expected additive ED_50_ values for pentazocine and neostigmine were 45.9 ± 3.9 *μ*g and 4.47 ± 0.43 *μ*g in phase I, respectively (Figure 4(a)). The expected additive ED_50_ values for pentazocine and neostigmine were 50.8 ± 3.5 *μ*g and 4.58 ± 0.42 *μ*g in phase II, respectively (Figure 4(b)). The experimental values for the pentazocine-neostigmine mixtures decreased significantly (*P* < 0.05) below the lines of additivity indicating a synergistic effect (Figures 4(a) and 4(b) and Table 1). The experimental ED_50_ for pentazocine and neostigmine were 34 ± 2.2 *μ*g and 3.3 ± 0.27 *μ*g, respectively (Figure 4(c)). The expected additive ED_50_ values for pentazocine and neostigmine were 51.0 ± 3.0 *μ*g and 4.85 ± 0.4 *μ*g, respectively (Figure 4(c)). The experimental values for the pentazocine-neostigmine mixtures decreased significantly (*P* < 0.05) below the lines of additivity, indicating a synergistic effect (Figure 4(c) and Table 1).

### 3.4. Intrathecal Antagonist Test

**Figure 5:**
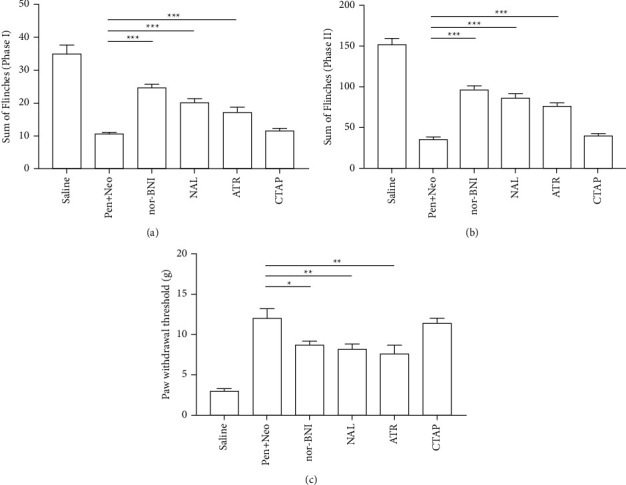
To evaluate the possible interaction mechanism between pentazocine and neostigmine, nor-Binaltorphimine (nor-BNI, 10 *μ*g), CTAP (10 *μ*g), naloxone (NAL10 *μ*g), and atropine (ATR 10 *μ*g), were intrathecally administered 30 minutes before the administration of the pentazocine (30 *μ*g)-neostigmine (3.0 *μ*g) mixture. (a) Nor-BNI, naloxone, and atropine significantly antagonized the antinociception of the pen-neo mixture in the first phase of formalin pain (One-way ANOVA, *F*_5,54_ = 36.437, *P* < 0.001). (b) Nor-BNI, naloxone, and atropine significantly antagonized the antinociception of the pen-neo mixture in the second phase of formalin pain (One-way ANOVA, *F*_5,54_ = 62.32, *P* < 0.001). (c) Nor-BNI, naloxone, and atropine significantly antagonized the antinociception of the pen-neo mixture in the plantar incision pain model (One-way ANOVA, *F*_5,54_ = 16.88, *P* < 0.001). ^*∗*^*P* < 0.05, ^*∗∗*^*P* < 0.01, ^*∗∗∗*^*P* < 0.001 vs. Pen + Neo group, *n* = 10 rats in each group. Each bar represents the sum of flinches in (a) phase (I), (b) phase II, and (c) the mechanical withdrawal threshold (mean ± SEM) from 10 rats.

We used the selective *κ*-opioid receptor antagonist nor-Binaltorphimine (10 *μ*g, nor-BNI), the selective *µ*-opioid receptor antagonist CTAP (10 *μ*g), the nonselective opioid receptor antagonist naloxone (10 *μ*g), and the muscarinic acetylcholine receptor antagonist atropine (10 *μ*g) to evaluate the possible synergistic effect mechanisms between pentazocine and neostigmine. Nor-BNI, naloxone, and atropine, but not CTAP, attenuated the analgesic effect of the pentazocine-neostigmine mixture against formalin-induced pain (Figures 5(a) and 5(b)) and the plantar incision-induced mechanical allodynia (Figure 5(c)).

## 4. Discussion

The results show that pentazocine and neostigmine produce analgesic effects against formalin-induced pain and incision-induced mechanical allodynia. There was a synergistic effect between pentazocine and neostigmine in both pain models. The formalin test was chosen as it is a valuable method for studying nociception in detecting drug analgesic effects [26]. The test showed a biphasic pain response. The early phase (0–6 minutes, Phase I) was mainly caused by the activation of C-fiber due to the peripheral stimulus, while the late phase (10–60 minutes, Phase II) was caused by an inflammatory reaction in the peripheral tissue and functional changes in the dorsal horn of the spinal cord [26]. In order to evaluate the analgesic effects of pentazocine and neostigmine in the postoperative period, we used the plantar incisional pain model [22].

As a mixed opioid agonist/antagonist, pentazocine's analgesic mechanism is not entirely understood. The spinal *µ*- and *κ*-opioid receptors are considered the most important pathways mediating the analgesic effects of pentazocine [27]. Different administration methods and dosages are also important factors that affect its analgesic actions. The analgesic mechanism of pentazocine varies with different doses and administrations [27]. Its analgesic action shows a biphasic bell-shaped dose-response curve. Intravenous injection of pentazocine at a modest dose (30 mg/kg) exhibits a peak antinociceptive effect via the *µ*- and *κ*-opioid receptors [27]. When an intravenous injection of pentazocine reaches a dose of 100 mg/kg, its analgesic effect is mainly through the *µ*-opioid receptor, not the *κ*-opioid receptor, as this analgesic effect can be partly antagonized by the *κ*-opioid receptor agonist [27]. *α*-adrenergic receptors might be other analgesic pathways of pentazocine as phentolamine alone is effective in reducing pentazocine's analgesic effects [28]. Nor-BNI is a highly selective kappa-opioid receptor antagonist that can partially antagonize the action of morphine and fentanyl [29]. In our results, the synergistic effects between pentazocine and neostigmine can be antagonized by the nonselective opioid receptor antagonist naloxone and the *κ*-opioid receptor antagonist nor-BNI, but not antagonized by the *µ*-opioid receptor antagonist CTAP. CTAP is a highly selective antagonist for *µ*-opioid receptors over *δ*- and *κ*-opioid receptors [30]. This shows that the combined medication of this dose is mostly via the *κ*-opioid receptor, but not the *µ*-opioid receptor, when intrathecally administered.

Systemic and spinal administration of acetylcholinesterase inhibitors and muscarinic receptor agonists can produce an analgesic effect [9, 12, 15]. Intrathecal injection of neostigmine and physostigmine produces dose-dependent antinociception effects and relieves allodynia in a dose-related manner [9, 12, 15]. The analgesic effect caused by neostigmine is mainly related to the release of acetylcholine and the activation of the muscarinic-acetylcholine receptor, as atropine blocks the muscarinic-acetylcholine receptor and antagonizes the analgesic effect [9, 12, 15]. However, when neostigmine is used as a nondepolarizing muscle relaxant antagonist, it is always used in combination with atropine as it antagonizes the muscarinic-acetylcholine receptor activity [13]. Normally, it inhibits acetylcholinesterase (AchE) and causes more ACh at sites of cholinergic transmission. ACh is released when physiological stimuli (pain) modulate the processing of pain at the spinal cord or brain stem level [11].

The administration of muscarinic receptor agonists and acetylcholinesterase inhibitors in the spinal cord can also result in antinociception [31]. The perioperative administration of physostigmine can reduce opioid consumption and peri-incisional mechanical allodynia [12]. Intrathecal neostigmine alone, or combined with clonidine, or opioids, has been successfully used for postoperative analgesic effects and pain relief [32], as it produces a longer effect with greater cardiovascular system reliability and fewer side effects. Epidural administration of neostigmine can prolong ropivacaine analgesia and reduce hourly ropivacaine consumption [33]. Intra-articular administration with a 500 *μ*g dose of neostigmine is as effective as a postoperative analgesic and is not likely to significantly increase the adverse effects [11]. The sustained analgesic effects of neostigmine after surgery are also interpreted as a decrease in the activation of the descending pathway of pain-induced acetylcholine release [34]. In our results, the synergistic effects between pentazocine and neostigmine were antagonized by the muscarinic acetylcholine receptor antagonist atropine, indicating that the muscarinic acetylcholine receptor might also be an important pathway for pentazocine-neostigmine's synergistic analgesic effect. The analgesic effect of intrathecal pentazocine and neostigmine may involve the pain descending inhibitory system. Pentazocine through the opioid and *σ*-receptor-independent pathway inhibits the norepinephrine transporter function and regulates the descending noradrenergic inhibitory system [35]. Previous works have identified that the spinal nicotinic acetylcholine receptors also affect pain regulation via the descending noradrenergic pathway [36]. We speculate that the descending inhibitory system might be another important pathway for the synergistic effect of pentazocine and neostigmine in formalin-induced pain and incision pain. Different doses of opioid receptor antagonists have different effects [37], and different types of opioid receptors show different effects in relieving thermal allodynia and mechanical pain [27]. This is the main research limitation of our work as we only used one antagonist dose and only two animal models.

We used isobolographic analysis to demonstrate the synergistic interaction between intrathecal pentazocine and neostigmine in both phases of the formalin test and the plantar incision model. There are several possibilities for this synergistic effect. Synergistic effect occurs when drugs have different effects at critical points along a common pathway [38]. The cholinesterase inhibitor modulates the transmission and processing of nociception according to the pre- and postsynaptic mechanisms, so simultaneous engagement of pre- and postsynaptic mechanisms may enhance the antinociception induced by either drug acting at one site independently [9, 16]. Moreover, two different receptors can simultaneously activate a common second messenger pathway in a single neuron and promote an effector mechanism [38, 39]. In this present study, the combined therapy of pentazocine and neostigmine produced a dose-dependent analgesic against formalin-induced pain and incisional mechanical allodynia. The combined use of pentazocine and neostigmine has a synergistic effect which may be related to the cholinergic system and the *κ*-opioid receptor at the spinal cord level.

## Figures and Tables

**Table 1:** ED_50_ and SEM for intrathecally administered pentazocine, neostigmine and in combination in a fixed-dose ratio.

Group	Drug	Pentazocine component		Neostigmine component		Sum of ED_50_ fractions
		Intrathecal dose (*μ*g)	Fraction of ED_50_	Intrathecal dose (*μ*g)	Fraction of ED_50_	
Formalin test Phase I	PEN	91.1 ± 5.4	1.00	----	----	
	NEO	----	----	8.93 ± 0.59	1.00	1.00
Formalin test Phase II	PEN	101.2 ± 6.2	1.00	----	---	
	NEO	----	---	9.20 ± 0.55	1.00	1.00
Plantar incision model	PEN	98.0 ± 5.5	1.00	----	----	
	NEO	----	----	10.2 ± 0.55	1.00	1.00
Interaction studies						
Formalin test Phase I	PEO + NEO	30.3 ± 2.5	0.33	2.95 ± 0.30	0.33	0.66
Formalin test Phase II	PEO + NEO	34.4 ± 3.0	0.34	3.10 ± 0.24	0.34	0.68
Plantar incision model	PEO + NEO	34.0 ± 2.2	0.35	3.31 ± 0.27	0.32	0.67

PEN = Pentazocine; NEO = Neostigmine.

## Data Availability

All the original data can be obtained from the corresponding author of the article by e-mail (ouyhd@sysucc.org.cn).
